# Ethno-pharmaceutical Formulations in Kurdish Ethno-medicine 

**Published:** 2014

**Authors:** Reza Tahvilian, Soheyla Shahriari, Akbar Faramarzi, Ayoob Komasi

**Affiliations:** a *Department of Pharmaceutics, Novel Drug Delivery Research Center, School of Pharmacy, Kermanshah University of Medical Sciences (KUMS) Kermanshah, Iran. *

**Keywords:** Ethno-pharmaceutical, Traditional medicine, Complementary therapy, Natural plants

## Abstract

Kermanshah is a city in west of Iran with a specific customs and cultures between the people who are living here. According to historical documents these cultures are very ancient and belong to more than one thousand years. The climate condition in this place forces people to find the solution of their problems using the plants and natural facilities. Therefore traditional healers were so active in Kermanshah. From 8000 of plant species in Iran more than 1200 species has grown in Kermanshah. The ancient customs, cultures, traditional medicine and formulations generally used by rural populations was transfer from ancient to modern people. Documentation of these traditional methods was studied in this research in order to compare and certified the traditional medicine with modern methods and find new dosage forms of drug with botanical source. It was established that about 50 plant species and 8 types of diseases were distinguished and cured by these people. It is also concluding that utilization of these plants approximately the same as application of plants in recent publications.

## Introduction

During the last decade, use of traditional medicine has expanded globally and has gained popularity. It has not only continued to be used for primary healthcare of the poor in developing countries, but has also been used in countries where conventional medicine is predominant in the national health care system. With the tremendous expansion in the use of traditional medicine worldwide, safety and efficacy as well as quality control of herbal medicines and traditional procedure-based therapies have become important concerns for both health authorities and the public (1). 

Traditional medicine has a long history. It is the sum total of the knowledge, skills and practices based on the theories, beliefs and experiences indigenous to different cultures, whether explicable or not, used in the maintenance of health, as well as in the prevention, diagnosis, improvement or treatment of physical and mental illnesses. The terms complementary/ alternative/non-conventional medicine are used interchangeably with traditional medicine in some countries (2). Traditional medicine is recognized as being important for safeguarding traditional livelihoods and supporting the well-being of people in all regions of the developing world (3, 4). According to World health organization (WHO), traditional medicine refers to “Health practices, approaches, knowledge and beliefs incorporating plant, animal, and mineralized medicine, spiritual therapies, manual techniques and exercises, applied singularly or in combination to treat, diagnose and prevent illnesses or maintain well-being” (5). The common wisdom is that poor and marginalized people are highly reliant on traditional medicine for their healthcare (6), but recent global quantitative estimates of the prevalence of the use of traditional medicine do not exist. In 1982, the WHO estimated that 80% of the world’s population relied exclusively or principally on traditional medicine for their healthcare (7).

More recently, increased attention has been focused on specific CAM therapies; including traditional East Asian medicine (8). Burke *et al., *studies showed that in comparing the two medicines, the patient samples in both countries were significantly more satisfied with Traditional Medicine (TM) than Alternative Medicine (AM) (8).

**Figure 1:**
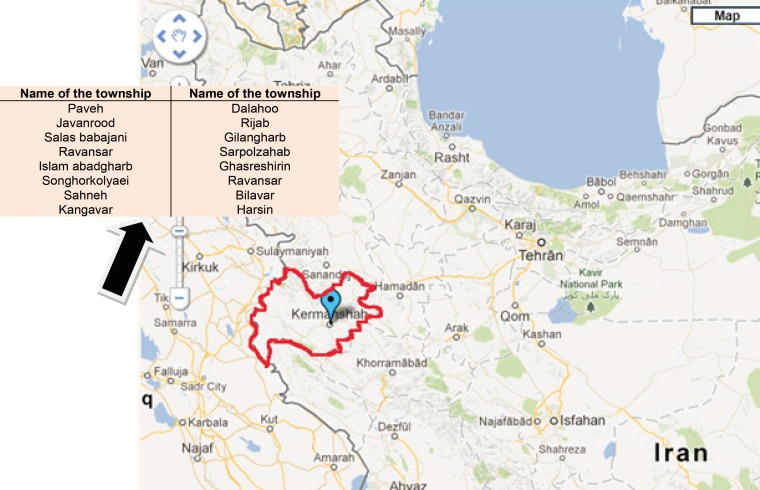
Study area map kermanshah Iran

The area of Kermanshah district is about 2463600 hectare located between North latitude 33º 36’ and 35º 15’ and between 45º24’ and 48 º 30’ East longitudes in west part of Iran (Figure 1). From 8000 of plant species in Iran more than 1200 species has grown in Kermanshah (9-13). The people who are living in Kermanshah in west part of Iran has different customs, cultures and climate conditions using traditional medicine, formulations generally are made by rural populations and transferring from ancient to modern people. According to historical documents these cultures are very ancient and belong to more than one thousand years (14). As the person to person information transferring is not relative, it is necessary to study different ancient medical procedure and document them. In this region medicinal plants are often the only easily accessible health care alternative for most of the population in rural areas and in fact folk herbal medicine is the most used remedy to cure common diseases. In this paper we present the most frequently used native species and the most common ethno-pharmaceutical preparations made from them, in order to preserve the plant popular knowledge, which has traditionally been only an oral one.

## Experimental

*Methods*

In this study, several field trips were undertaken to different localities of rural and urban community in Kermanshah. During the survey, the plants medicinal knowledge about usage and treating various diseases were gathered from local healers via questionnaires and interviews Table 1. The snowball sampling method helps to find more relevant ancient healers. The traditional formulations, consumed materials and natural plants or animal components were registered in order to encourage them to give procedure of product preparation in detail.

Different type of disease and plant materials used in these methods and formulations were categorized and compared with modern scientific information.

**Table 1:** The sample of questionnaire

In the name of God			
Name:	Family:	Job:	Education level:
job history:	Age:	Address:	gender:
What is the name of your therapeutic method? What are the constituents of your formulation? If you have any type of plants in your formulation please declare the name of plant, parts of used and the time of harvesting and the method of herbal preparation? What is the method of making traditional preparation and storage? What are the application methods? What are the traditional indications of your preparation? Have seen any side effects from this preparation? Does your preparation have any interaction with other chemicals or natural materials? What is your advice for duration of drug application? What are the results of drug application for your formulation? How many people have used this formulation until now? Would the subjects like to use this formulation again?			












After data gathering it will be possible to evaluate the registered information in comparison to modern therapeutic methods by making contemporary dosage forms. It is also possible to modify these formulations and verify the clinical effects by special physicians in the future. The plants used in the Kurdish ethno-medicine were identified by using floristic, taxonomic references in Agricultural and Natural Resources Research Center, Kermanshah (Iran).

## Results

**Figure 2:**
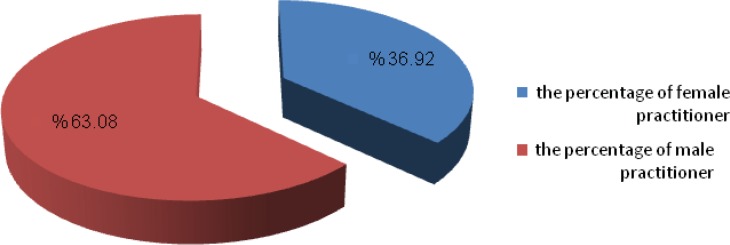
The sex distribution of practitioners

The results collected from about130 traditional physicians in 70 rural and district show in the following tables (Tables 2, 3). As it is showing in the Figure 2, 48 person of practitioner were female and the other healers were male. The distribution of different practitioner with different ages has shown in Figure 2. It was established that a large number of them had more than 50 years old. It is established that there is no affinity in young people in these rural to learn and also know about traditional medicine and also the ethno pharmaceutical formulation preparation.

**Table 2:** Plants consumed in Kermanshah province, along with ethno-medicine formulations

**The consumption**	**Dosage form**	**Traditional application for clinical symptoms and diseases**	**Parts used**	**Common name**	**Family name**	**Scientific name**	**Row**
Oral	Infusion	Dissolves renal calculi	Leaf	Ghitaran	Pteridaceae	*Adiantum capillus-veneris* L.	1
Oral	Infusion	Abdominal pain control					
Oral,	Infusion	Dysuria relief					
Oral	Decoction	Bechic					
Oral	Decoction	Reduce blood cholesterol	Whole plant	Koul	Alliaceae	*Alium colchicifolium *Boiss.	2
Oral	Soup	Laxative	Leaf	Ghaz or Haz	Araceae	*Arum conphalloides *Ky.exschott	3
Oral	Soup	Anti helmintic					
Oral	Decoction	Hypotensor					
Oral	Soup	Tonic for pregnant women					
Oral	Decoction	Dissolves renal calculi,	Fruit	Gochaneh-gia	Leguminosae	*Astragalus hamosus* L.	4
Inhalation	Moist fumigation	Headache relief					
Oral	Decoction	Anti gastric,					
Vaginal	Moist fumigation	Treatment of vaginitis					
Topical	Fresh paste	Analgesic	A: Root B: Fruit	Kalak-Maraneh	Capparaceae	*Capparis spinosa* L.	5
Oral	Powder	Diabetes control ,					
Oral	Decoction	Dissolves renal calculi					
Oral	Decoction,	Laxative,	Leaf	Ghenavleh	Brassicaceae	*Cardaria draba* (L.) Desv	6
Topical	Plaster	Anti-headache,					
Oral	Soup	Anti gastric					
Topical	Paste	Burn healing	Aerial parts	Zarde-siri	Asteraceae	*Carthamus oxyacantha* M.B	7
Oral	Decoction	Diabetes control	Seed	Meroor	Dipsacaceae	*Cephalaria dichaetophora *Boiss.	8
Oral,	Powder,	Diabetes control	Seed	Geshnij	Apiaceae	*Coriandrum sativum* L.	9
Oral	Decoction,	Carminative					
Oral	Decoction	Gout control					
Topical	Fresh paste	Burn healing,	Root	Zou	Apiaceae	*Dorema aucheri* Boiss.	10
Topical	Fresh paste	Cornicide					
Oral	Sweat	Dissolves renal calculi,	Aerial parts	Keravi	Apiaceae	*Echinophora platyloba* DC.	11
Oral	Infusion	Bechic,					
Mouth wash	Decoction	Anti aphthous					
Smoke spread in the environment,	Dry fumigation	Antiseptic environment	Aerial parts	Shir-khoshi	Ehphorbiaceae	*Euphorbia helioscopia* L.	12
Oral	Fresh latex	Purgative					
Oral	Decoction	Carminative	Leaf	Paghaze	Apiaceae	*Falcaria vulgaris *bernh.	13
Oral	Decoction	Febrifuge					
Topical	Powder	Vulnerary					
Oral	Decoction	Stomachic					
Topical	Powder	Hemostatic					
Oral	Powder	Oil preservative	Aerial parts	Chenour	Apiaceae	*Ferulago angulata* (schlecht.) Boiss.	14
Topical	Powder	Cure wounds	Bulb		Liliaceae	*Fritillaria imperialis* L.	15
Vaginal	Decoction	Treatment of vaginitis	Root	Balak Common name	Leguminosae	*Glycyrrhiza glabra *L.	16
Oral	Decoction	Quit smoking					
Oral	Decoction	Anti-ulcer,					
Mouth wash	Decoction	Anti-aphthous					
Topical	Paste	Burn healing	Root	Ghenger	Asteraceae	*Gundeliato urnefortii* L.	17
Topical	Dry fumigation,	Anti-eczema,	Aerial parts	Barazha	Solanaceae	*Hyoscyamus niger* L.	18
Topical	Ointment	Burn healing					
Oral	Decoction	Dissolves renal calculi	A: Leaf B: Root	Baraza	Umbelliferae	*Johernia aromatic *Rech. F.	19
Topical	Decoction	Cornicide					
Inhalation	Moist fumigation	Anti-migraine	Aerial parts	Now sake	Lamiaceae	*Marrubium cuneatum* Russell.	20
Topical	Fresh paste	Vulnerary	Aerial parts	Shaoudar	Fabaceae	*Melilotus officinalis* Lam.	21
Oral	Infusion	Dissolves renal caculi					
Oral	Decoction	Anti-histamine and anti-pruritus	Aerial parts	Fatmah darou	Lamiaceae	*Melissa officinalis* L.	22
Oral	Decoction	Diabetes control					
Oral	Powder	Anti-diarrhea	Aerial parts	Ponah	Lamiaceae	*Mentha longifolia* (L.) Hudson.	23
Oral	Decoction	Abdominal pain control					
Oral	Decoction	Pectoral					
Oral	Decoction	Treatment of vaginitis	Leaf	Kouzalah	Brassicaceae	*Nasturtium officinale (L.) R. Br.*	24
Oral	Decoction	Galactogogue	Seed	Siya-sonoy	Ranunculaceae	*Nigella sativa *L.	25
Topical	Powder	Anti-hyperpigmentation					
Topical	Powder	Anti-scar					
Rectal	Ointment	Anti-hemorrhoid	Flower	Kar-koul	Compositae	*Onopordon heteracanthum* C. A. Mey	26
Topical	Paste	Anti-spot					
Oral	Decoction	Hypotensor					
Oral	Juice	Dissolves renal caculi					
Topical	Ointment	Burn healing	Root	Asalak	Boraginaceae	*Onosma rostellatum *Lehm.	27
Topical	Ointment	Vulnerary					
Topical	Extract mixed with yogurt	Anti-acne	Flower	Kasa-shekan	Papaveraceae	*Papaver rhoeas* L.	28
Oral	Infusion	Bechic					
Ophthalmic drop	Infusion	Ocular anti inflammatory					
Oral	Infusion	Anti-cold					
Smoke spread in the environment (air)	Dry fumigation	Antiseptic environment	Seed	Espan	Zygophyllaceae	*Peganum harmala* L.	29
Topical	Inspissated juice	Hemostatic					
Topical	Soft extract	Vulnerary	Leaf	Gobarekhe	Lamiaceae	*Phlomis olivieri *Benth.	30
Vaginal	Suppository	Overcoming infertility in women	Leaf		Plantaginaceae	*Plantago lanceolata* L.	31
Topical	Fresh paste	Analgesic	Leaf	Hara-kishah	Plantaginaceae	*Plantago major *L.	32
Topical	Powder	Vulnerary					
Topical	Fresh paste	Maturative					
Oral	Decoction	Gout control	Seed	Degan-tijkar	Portulacaceae	*Portulaca oleracea* L.	33
Oral	Decoction	Anti-acne					
Oral	Fresh juice	Dissolves renal caculi	Aerial parts	Revas	Polygonaceae	*Rheum ribes *L.	34
Oral	Decoction	Rheumatic pains control					
Oral	Powder	Anti-diarrhea					
Oral	Powder mixed with	Burn healing	Flower	Tourshakeh	Polygonaceae	*Rumex elbursensis *Boiss.	35
Oral	yogurt, Fresh fruit	Diabetes control					
Oral	Decoction, Soft	Anti-diarrhea					
Oral	Extract	Anti-ulcer					
Oral	Decoction	Bechic	Leaf	Tourshak	Polygonaceae	*Rumex ephedroides *Bornm.	36
Topical	Decoction	Vulnerary	Aerial parts	Zengla-bechek	Scrophulariaceae	*Scrophularia striata *Boiss.	37
Topical	Decoction	Burn healing					
Oral	Decoction	Anti-ulcer					
Topical	Decoction	Anti- dandruff					
Oral	Decoction	Bechic					
Topical	Ointment	Burn healing	Seed	Konji	Pedaliaceae	*Sesamum indicum* L.	38
Oral	Decoction	Anti- helmintic	Root	Gonour	Apiaceae	*Smyrnium cordifolium *Boiss.	39
Skin contact with the smoke	Dry fumigation	Anti- eczema	Fruit	Rezleh	Solanaceae	*Solanum nigrum* L.	40
Mouth contact with the smoke	Dry fumigation	Anti-toothache					
Oral	Infusion	Carminative	Aerial parts	Goula-chay	Lamiaceae	*Stachys lavandulifolia *Vahl.	41
Oral	Infusion	Abdominal pain control					
Oral	Decoction	Dysuria relief					
Oral	Decoction	Anti- diarrhea					
Oral	Powder	Digestive	Leaf	Sheng	Asteraceae	*Tragopogon collinus* DC.	42
Oral	Decoction	Anti-ulcer	Aerial parts	Azbovah	Lamiaceae	*Thymus kotschyanus* Boiss.et Hohen	43
Vaginal	Moist fumigation	Treatment of vaginitis					
Oral	Decoction	Bechic					
Oral	Decoction	Dissolves renal caculi	Fruit	Pey-kol	Zygophyllaceae	*Tribulus terrestris* L.	44
Oral	Fresh juice	Treat neonatal jaundice	Aerial parts	Shoudar	Fabaceae	*Trifolium repens *L.	45
Oral, Oral	Powder, Decoction	Burn healing, Calamative	Seed	Shemlieh	Fabaceae	*Trigonella monatha* G. A. Mey	46
Nasal drop	Fresh juice	Control of nasal bleeding	Leaf	Gazanah	Urticaceae	*Urtica dioica* L.	47
Oral	Decoction	Diabetes control					
Rectal	Soft extract	Anti-hemorrhoid					
Oral	Decoction	Diabetes control	Fruit	Gayanah	Fabaceae	*Vicia sativa *L.	48
Topical	Paste	Anti-acne	Fruit	Mowkherr	Viscaceae	*Viscum album *L.	49
Topical	Powder	Vulnerary	Leaf and Flower	Azbovah	Lamiaceae	*Ziziphora cliniopodioides *Lam.	50
Ophthalmic drop	Decoction	Ocular anti inflammatory					
Inhalation	Moist fumigation	Anti-headache					
Oral	Decoction	Anti-diarrhea					
Oral	Decoction	Calamative					

**Table 3:** Ethno-pharmaceutical formulations in Kermanshah traditional medicine

**The consumption**	** Forms of drug**	**Formulation components **	** Illness**	**Row**
Oral Oral	Mixed Decoction	A:*Punica granatum *L. fruit powder *Pistacia mutica *Fisch.et My. gumtree *Quereus persica *J.&SP fruit powder White tragacant Honey B: *Falcaria vulgaris *bernh. leaf *Astragalus hamosus* L. fruit *Tribulus terrestris* L. fruit *Ziziphora cliniopodioides* Lam. aerial parts *Glycyrrhiza glabra* L. root *Alcea *spp.flower	**Ulcer**	1
Inhalation Topical	Moist fumigation Juice	A: *Nerium oleander *L. leaf *Salix alba *L. leaf *Persica vulgaris* L. leaf B: *Apium petroselinum* L. leaf *Ocimum basilicum* L. leaf * Allium cepa* L. bulb	**Headache**	2
Oral Oral Oral	Mixed Decoction Mixed	A: *Quereus persica *J.&SP fruit powder *Vitis* spp. leaf powder *Rhus coriara*L. fruit powder Eggshell powder Yoghurt B: *Mentha longifolia *(L.) Hudson. aerial parts *Stachys lavandulifolia *Vahl. leaf *Ziziphora cliniopodioides* Lam. leaf *Matricaria chamomilla* L. flower Candy C: *Mentha longifolia *(L.) Hudson. aerial parts *Pistacia mutica *Fisch.et My. unripe fruit powder *Punica granatum *L. fruit powder Yoghurt	**Diarrhea**	3
Oral	Decoction	A: *pistaciamutica*Fisch.et My.Leaf *Cerasus microcarpa *(C.R.Mey) Boiss. leaf *Rosa canina* L. fruit *Capparis spinosa* L. fruit *Crata gus pseudoheterophylla *Pojark. fruit *Juglans regia *L. leaf	**Diabetes**	4
Oral Oral	Decoction Juice	A: *Astragalus hamosus *L. fruit *Capparis spinosa* L. fruit *Tribulus terrestris * L. fruit *Echinophora platyloba* DC. aerial parts *Melilotus officinalis *Lam. aerial parts B:*Rheum ribes* L. aerial parts *Onopordon heteracanthum* C. A. Mey. flower *Crataegus pseudoheterophylla *Pojark. leaf *Johernia aromatica *Rech. F. leaf	**Renal caculi**	5
Topical Topical Topical	Decoction Mixed Mixed	A: *Falcaria vulgaris *bernh. Leaf *Scrophularia striata *Boiss. aerial parts *Alcea *spp.flower *Quereus persica *J.&SP leaf *Rubus sanctus *L. leaf & root *Amygdalus eburnean *spach. Leaf B: *Falcaria vulgaris*bernh. leaf powder *Plantago major *L. leaf powder *Pistaciamutica*Fisch.et My. gumtree Bee wax Rump C:* pistacia mutica *Fisch.et My. gumtree *Ziziphora cliniopodioides* Lam. aerial parts powder *Matricaria chamomilla* L. flower powder *Smyrnium cordifolium *Boiss. root powder Honey	**Wound**	6
Topical Topical Topical	Ointment Ointment Mixed	A: *Scrophularia striata *Boiss.. aerial parts *Onosmaro stellatum *Lehm. root *Rubus sanctus *L. leaf & root *Pistaciamutica*Fisch.et My. gumtree Butter B: *Onosmarostellatum*L. Root *Pistacia mutica *Fisch.et My. gumtree *Alcea *spp.flower White tragacant Bee wax Butter C:*Sesamumindicum* L. fruit *Quercus infectoria *Oliv. galle powder *Hordeum vulgare* L. ash *Zizyphus vulgari*s Lam. Fruit Yoghurt	**Burn**	7
Vaginal Vaginal Vaginal	Suppository Suppository Moist fumigation	A: *Eryngium thyrosoideum *Boiss. Root * Phoenix dactylifera *L. fruit Rump B: *Plantago laonceolata* L. * Crocus sativus* L. Rump C: *Nasturtium officinale* (L.) R. Br. aerial parts *Ziziphora cliniopodioides* Lam. Leaf *Ulmus carpinifolia *Gleditsch.	**Infertility**	8

According to the above tables it was established that in the traditional treatment in Kermanshah the most popular types of dosage forms that made by practitioner is restricted to some topical and oral dosage forms. But in some instances it is very important that refer to some specialized dosage forms like vaginal suppository and some elementary inhalants. It was established that about 50 plant species and 8 types of diseases and symptoms were distinguished by these people.

The types of diseases that were treated in traditional medicine are related to some simple diseases that possible to distinguish or problems caused by trauma.

According to the new investigations some of these plants with most consumption in these places are certified in modern or traditional Iranian medicine that some of these plants are discussed at the following:

*Punica granatum L. or pomegranates*

This plant is cultivated in the west part of Iran and used in the Kurdish traditional medicine as an anti-diarrhea and ulcer healer. It was used in different preparation mixed with other plants for treating the above diseases in oral dosage form. In modern medicine it is also used as a proper plant for removing the bacterial and fungal infections as a mouthwash (15, 16).The presence of active constituents like tannic acid or alkaloids certifies that it could be useful as an anthelmintic or the antiviral drug (17). Other Investigators have established that, Juice consumption may also inhibit viral infections while pomegranate extracts have antibacterial effects against dental plaque (18-20). Table 3 showed that in traditional Kurdish medicine, pomegranate application for treating ulcer may be related to its antibacterial and anti-fungal effects which has also established in modern medicine. The anti-diarrheal effect of this plant is also similar between traditional Kurdish and modern medicine because the presence of the tannins could have an important role in diarrhea prevention (row 1and 3 Table 3).

*Glycyrrhiza glabra L. or Liquorice*

This is a self-grown plant medicine in the area under the investigation in Kermanshah. Usually the farmers get rid of a large amount of this herb as weeds. Liquorice grows best in deep valleys, well-drained soils, with full sun, and is harvested in the autumn, two to three years after planting (21).

This plant was used in the treatment of diseases and symptoms like vaginitis, Quit smoking, Anti-ulcer, Anti-aphthous in local traditional medicine. These findings are in accordance with the modern medicine. Recent studies indicate that glycyrrhizic acid disrupts latent Kaposi›s sarcoma (as also demonstrated with other herpesvirus infections in the active stage), exhibiting a strong anti-viral effect. The Chinese use liquorice to treat Tuberculosis. It was reported that liquorice inhibits *Helicobacter pylori*; therefore, it is used as an aid for healing stomach and duodenal ulcers and in moderate amounts may soothe an upset stomach. Liquorice can be used to treat ileitis, leaky gut syndrome, irritable bowel syndrome and Crohn›s disease as it is antispasmodic in the bowels (22-24).

*Plantago major L. («broadleaf plantain» or «greater plantain»)*

Plaintain is found all over the world, and is one of the most abundant and accessible medicinal herbs (25). It contains many bioactive compounds, including allantoin, aucubin, ursolic acid, flavonoids, and asperuloside (26-28). Scientific studies have shown that plantain extract has a wide range of biological effects, including wound healing activity, anti-inflammatory, analgesic, antioxidant, weak antibiotic, immuno modulating and antiulcerogenic activity (28).In this study the medicinal effects of plaintain was used for wound treating. It is obvious that some types of plantago local applications are compatible with modern medicine. 

*Juglans regia *L. the constituents of this plants are quinones, oil, tanin, fatty acids like cis- linoleic acid and linoleic acid. It is also contains folic acid, furural, einositol, Juglone, triptophan, catechictanins and flavonoides derivatives like hyperoside and jouglanin, and vitamin C. According to the presence of the above constituents, anti-fungal, antimicrobial, insecticide, anti-tumor and weeds growth inhibition effects have been established for this plant (29, 30).In modern medicines similar to traditional medicine in Kermanshah, the preparations prepared from leafs of juglans showed an anti-diabetic effects (30).

*Quercus Spp. Or Oak*

According to modern investigation, Oak has shown good effects on viral and bacterial infections. It has also shown a proper application in wound healing (31-33). As it is mentioned in the Table 3, this plant is used in burn treating as a wound healing and antibacterial agent in traditional medicine in Kermanshah province.

## Discussion

**Figure 3:**
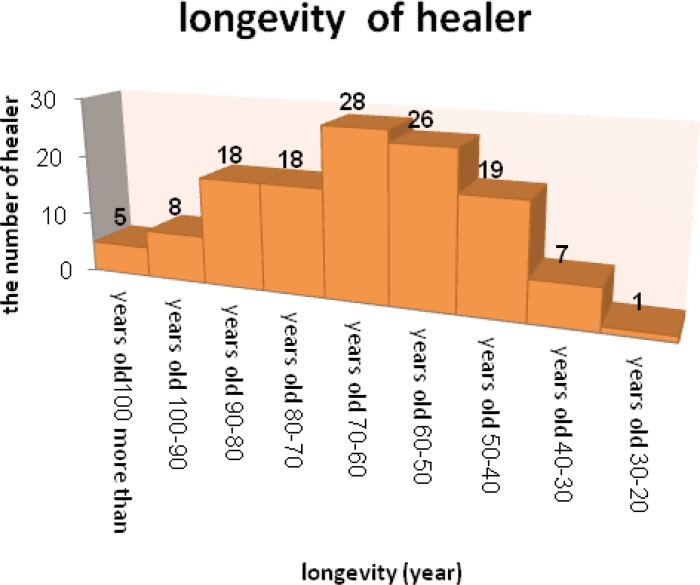
The longevity of practitioner

As it is shown in Figure 3 the maximum number of healer has more than 50 years old. This is proved that there is no affinity in young people in these rural to learn and also know about traditional medicine and also the ethno pharmaceutical formulation preparation. Therefore it is necessary to continue studies like this research and document different type of ethno pharmaceutical formulations. This matter is similar to the results of other researches about the ethno-pharmaceutics in other places in Iran. Abdolbaset Ghorbani, were established that there is same problems in documenting the ethno-pharmaceutical formulations in Turkeman society (34).With changes in the environment and life conditions it is common that in most of the ethno-botanical works informants believe that more medicinal plants were in use in past than now (35) and this work is no exception in this regard. This is as a result of the modern care system expansion and using synthesized medicines. Also the continued environmental degradation of medicinal plant habitats has brought the depletion of medicinal plants and the associated knowledge. Knowledge of medicinal plants is disappearing because most of the people with medicinal plant knowledge are passed away without properly passing their knowledge to the next generations. Today there are few professional healers (Tebibs) in the area, which regularly serve the community. Most of the knowledge of medicinal plants is owned by elders, who use the plants for their own families. Also elder women and traditional midwifes have important role in keeping home remedies, but they have fear to use their knowledge for the other families because the modern medical care system has banned them from using these practices. Unlike to Ghorbani›s investigation in this work, we could properly record the knowledge of women because there was no any problem to get information from woman›s healers (34). Unlike to other studies the common name of different plants in the rural and villages of Kermanshah are different and in some cases the plants with the same name had different common name and vice versa. Therefore, after sampling we tried to find the scientific name of the plants which mentioned in Table 2. 

The most interesting point that exploited from the healers responses to the questionnaire, was that the reported side effects from those therapeutic methods were seldom. They also mentioned that, they haven’t seen any interaction with other materials and they were suggesting confidentially those methods to patients. In some cases they were some volunteers that concerned to continue the treatment. The number of people who used the ethno pharmaceutical formulations was more than thousands of subjects. 

Although, according to novel methods of medicinal treatment, all of these procedures are not fully acceptable, but it is necessary to start investigating on the evaluation of these formulations on different type of diseases, using modern procedure of clinical trials and laboratory instruments in order to established or reject the efficacy of these therapeutic methods.
